# Successful Transition From Covert, Involuntary Oral Medication to Consensual, Long-Acting, Injectable Antipsychotic Therapy in a Patient With Schizophrenia: A Case Report From Japan

**DOI:** 10.7759/cureus.91904

**Published:** 2025-09-09

**Authors:** Yoshiyo Oguchi, Nobumi Miyake

**Affiliations:** 1 Department of Neuropsychiatry, St. Marianna University School of Medicine, Kanagawa, JPN; 2 Department of Neuropsychiatry, Kawasaki Tama Municipal Hospital, Kawasaki, JPN

**Keywords:** caregiver burden, covert medication, ethical dilemma, japanese psychiatry, long-acting injectable, medication non-adherence, paliperidone palmitate, personal recovery, schizophrenia, shared decision-making

## Abstract

Medication non-adherence is a primary obstacle in schizophrenia management, often leading to the involuntary or covert administration of medication by caregivers. Covert medication, the practice of administering medicine to a person without their knowledge, occurs in Japan within a complex ethical and legal landscape that lacks clear institutional frameworks, posing significant dilemmas for patients, their families, and clinicians. This report describes a case of a successful transition from such treatment to consensual, patient-centered therapy using long-acting injectable (LAI) antipsychotics. A 57-year-old Japanese man with a long history of schizophrenia experienced repeated relapses due to poor adherence to medication. For three months, his aged father covertly administered oral risperidone. This ethically unsustainable situation prompted a therapeutic shift. After engaging in shared decision-making focused on the patient’s personal recovery goals, such as improving computer skills and living more independently, he consented to therapy with paliperidone palmitate LAI. The transition to LAI therapy resulted in marked clinical and functional improvements, evidenced by improved scores across all domains of the Brief Evaluation of Psychosis Symptom Domains and a significant increase in his Global Assessment of Functioning score. The patient resumed household tasks, participated in community activities, and expressed aspirations for the future, leading to dramatic improvements in quality of life and a substantial reduction in caregiver burden. This case highlights how LAIs can serve as a vital tool to resolve the ethical challenges of covert medication, respect patient autonomy, and align with principles of personal recovery. LAI antipsychotics offer an effective and ethically sound alternative to covert medication in schizophrenia. This approach can strengthen the therapeutic alliance, enhance patient autonomy, and lead to meaningful improvements in clinical symptoms and real-world functioning, providing a valuable solution within challenging sociocultural contexts.

## Introduction

Schizophrenia affects approximately 1% of the global population and is characterized by positive and negative symptoms as well as cognitive deficits that significantly impact social functioning and quality of life (QoL) [1,2]. Poor adherence to medication is a primary challenge in its management [3]. In cases of severe non-adherence, families may resort to covert medication - the administration of medication without the patient’s knowledge; for instance, by mixing it with food or drink [4]. This practice, often a last resort for desperate caregivers, is a significant issue. A cross-sectional study in India found that approximately one-third of patients with severe mental illnesses had received medication this way at some point [4].

This issue is particularly complex in Japan. While international ethical guidelines, such as those from the World Health Organization (WHO), unequivocally prioritize patient autonomy and informed consent in mental healthcare [5], the practice of covert medication exists in a legal and ethical “gray zone” in Japan. Although legally prohibited in principle, Japan lacks adequate legal and institutional systems to guide clinicians and families in such circumstances [6]. This gap between principle and practice is exacerbated by sociocultural factors, most notably the “8050 problem” - a growing social crisis in Japan where elderly parents (in their 80s) are the primary caregivers for their socially withdrawn, often mentally ill, adult children (in their 50s) [7]. A 2023 Japanese Cabinet Office survey estimated this *hikikomori* population (people with severe social withdrawal) to be approximately 1.46 million, underscoring the scale of this societal challenge [8]. The immense burden on these aging caregivers often leads to situations of covert medication, creating significant ethical challenges and highlighting the urgent need for therapeutic alternatives that uphold patient autonomy.

Long-acting injectable (LAI) antipsychotics represent one such alternative. LAIs are administered via intramuscular injection at intervals ranging from two weeks to three months, providing sustained therapeutic plasma concentrations of the medication. This mode of delivery ensures treatment continuity, circumvents the need for daily oral intake, and has been shown to improve adherence and reduce relapse rates compared to oral antipsychotics [9]. However, LAIs remain underutilized in Japanese psychiatric care, with prescription rates below 5%, in part due to psychiatrists' negative attitudes regarding issues such as cost, injection pain, and perceived patient refusal [10].

We report the case of a middle-aged man with schizophrenia who, after years of poor adherence and repeated relapses, received covert oral antipsychotic treatment from his aged father. This report describes his successful transition to consensual long-acting injectable (LAI) therapy, offering a practical and ethical solution to a common clinical dilemma deeply rooted in the Japanese sociocultural context.

## Case presentation

A 57-year-old male with no family history of psychiatric disorders began experiencing auditory hallucinations and thought broadcasting at the age of 24. He graduated from university but has never been employed, living with his parents in the family home his entire adult life. His mother passed away recently, and his elderly father is his sole caregiver and social support. He was diagnosed with schizophrenia but exhibited persistent treatment refusal, rarely engaging in his appointments. After his first hospitalization at age 31, he was treated with olanzapine but remained socially withdrawn with unstable symptoms due to poor insight, leading to multiple subsequent hospitalizations.

He was seen at the outpatient psychiatric clinic of St. Marianna University School of Medicine Hospital. At his first outpatient visit, accompanied by his aged father, the patient presented with a marked loosening of associations. Five years later, his father brought him back due to concerns about loud vocalizations and bizarre behavior. The patient refused olanzapine but tolerated oral paliperidone (3 mg). Four months later, his father reported difficulties in administering the medication and requested liquid risperidone, which was subsequently prescribed.

For approximately three months, the father administered the liquid risperidone covertly by mixing it into the patient’s food. The treating physician was unaware of this. The practice came to light when the father, during a consultation, confessed his actions, stating he was overwhelmed and that this approach was no longer sustainable. Confronted with this ethically untenable situation and the father’s own impending hospitalization for health issues, we arranged for the patient to be admitted for treatment stabilization and care planning.

During the hospitalization, as the patient’s psychotic symptoms (e.g., auditory hallucinations) subsided and his insight into his condition partially improved, we engaged him in a conversation about his future aspirations. This was facilitated using principles of motivational interviewing and shared decision-making. We first explored his life goals before discussing treatment. He expressed desires to “improve computer skills,” “manage household tasks,” and “live authentically,” while revealing his primary reason for non-adherence: “daily medication feels like admitting illness.”

This crucial insight allowed us to frame LAI therapy not as a treatment for his illness, but as a tool to achieve his goals. We proposed it as an alternative that would free him from the daily reminder of his condition. The consent process was a multidisciplinary effort: the physician (Y.O.) explained the benefits (stable symptom control, no need for daily pills) and risks (e.g., injection site pain), a clinical nurse provided information on the injection procedure, and a psychiatric social worker explained how the national health insurance system would reduce the financial cost. With his father’s encouragement, the patient provided informed consent to this new approach. He was initiated on paliperidone palmitate LAI (150 mg, followed by 100 mg) and was discharged on a regimen of 75 mg monthly injections. For the first time, visiting nurses were successfully introduced to provide community-based support.

The transition to LAI therapy resulted in significant clinical and functional recovery. The patient experienced no notable side effects from the LAI therapy, including no extrapyramidal symptoms or significant injection site pain.

**Figure 1:**
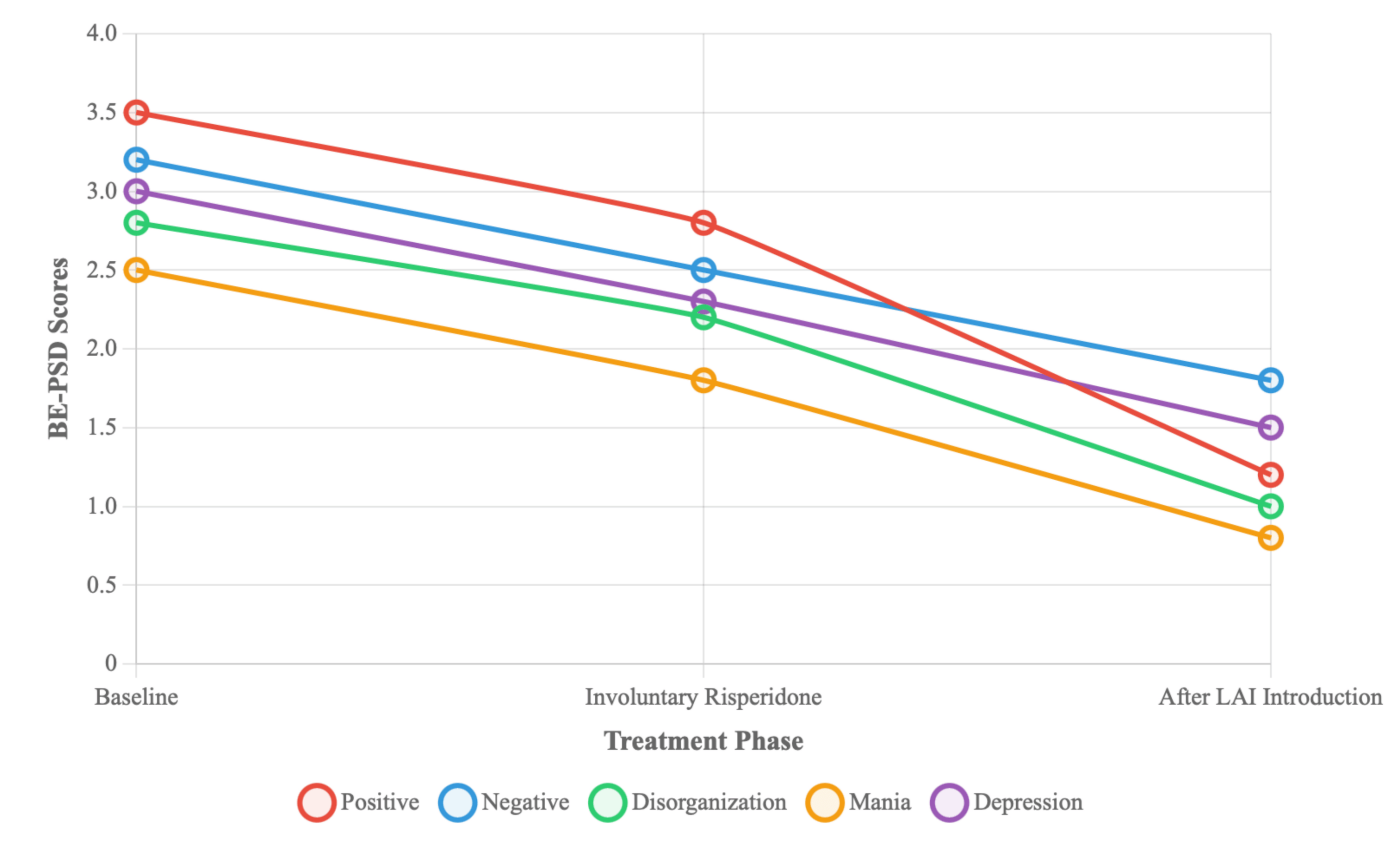
Changes in Brief Evaluation of Psychosis Symptom Domains (BE-PSD) scores over the course of treatment Scores for the five symptom domains (positive, negative, disorganization, mania, and depression) are plotted across three distinct treatment phases: at baseline (before intervention), during covert administration of oral risperidone, and after the transition to consensual long-acting injectable (LAI) paliperidone therapy. Each line represents a distinct symptom domain, illustrating the trajectory of improvement over time. Lower scores indicate a reduction in symptom severity.

Symptom changes were assessed using the Japanese version of the Brief Evaluation of Psychosis Symptom Domains (BE-PSD), with permission from the scale developer, Dr. Hiroyoshi Takeuchi [11]. Scores at baseline, during covert treatment, and after LAI introduction were: Positive (3.5, 2.8, 1.2), Negative (3.2, 2.5, 1.8), Disorganization (2.8, 2.2, 1.0), Mania (2.5, 1.8, 0.8), and Depression (3.0, 2.3, 1.5). These scores showed substantial improvement across all domains following the introduction of LAI therapy (Figure 1).

**Figure 2:**
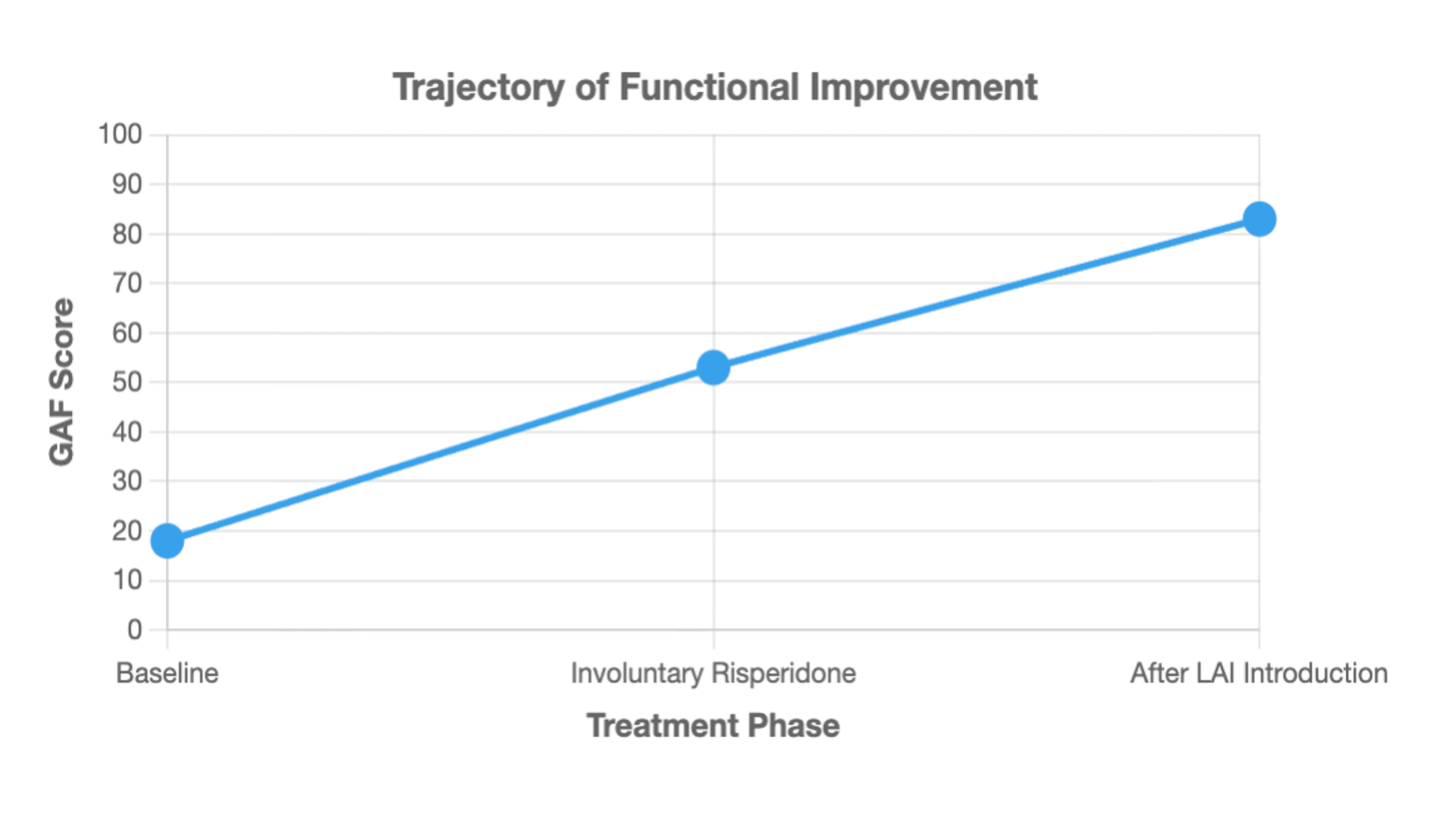
Changes in Global Assessment of Functioning (GAF) score over the course of treatment. The GAF score is plotted across three distinct treatment phases: at baseline (before intervention), during covert involuntary administration of oral risperidone, and after the transition to consensual long-acting injectable (LAI) paliperidone therapy. Higher scores indicate superior overall functioning.

Functional improvement was measured using the Global Assessment of Functioning (GAF) scale. The patient’s GAF score improved from 18 at baseline (indicating a need for constant supervision) to 53 during the covert risperidone phase (moderate difficulty in social functioning), and finally to 83 after 10 months of LAI therapy (symptoms are transient and expectable reactions to psychosocial stressors), representing a dramatic recovery in real-world functioning (Figure 2).

**Figure 3:**
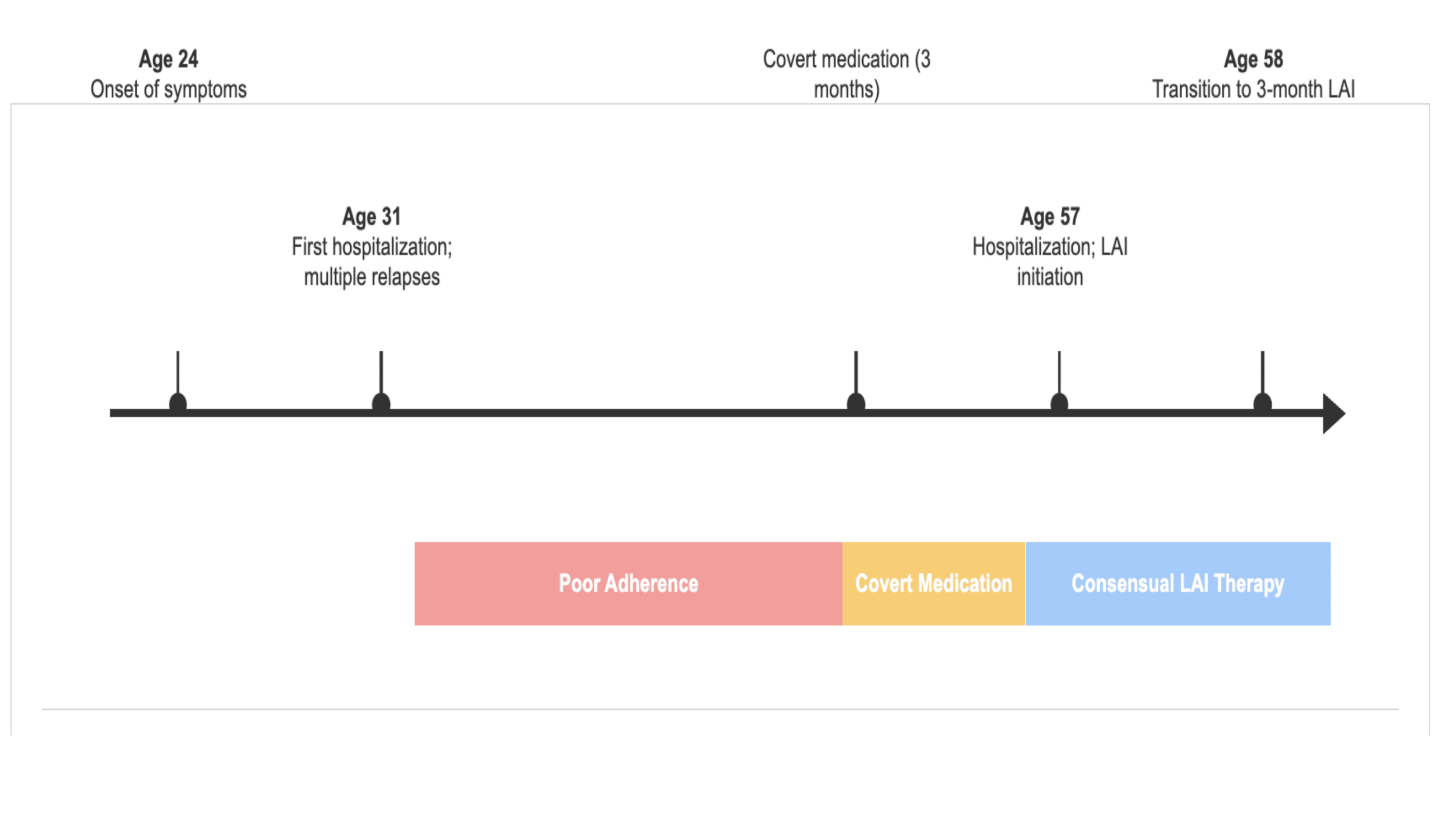
Timeline of the patient’s clinical course, key interventions, and treatment phases The upper portion marks significant clinical events from the onset of symptoms to the transition to a three-month LAI formulation. The lower colored bars represent the three distinct therapeutic phases: a prolonged period of poor adherence, a three-month period of covert medication by the caregiver, and the subsequent successful transition to consensual LAI therapy. LAI, long-acting injectable

The patient and his recovered father attended follow-up appointments together. The father reported, “He’s stable and we can shop together, different from oral medication times.” The patient gradually resumed household tasks and began attending computer classes. After 10 months of stable treatment, he and his father welcomed the transition to a 3-month formulation (263 mg), which was initiated at month 11. He has since remained stable for over a year, attends appointments independently, and has recently expressed a desire to get married. The patient’s clinical course is summarized in Figure 3.

## Discussion

This case illustrates a successful transition from covert, involuntary oral medication to consensual LAI therapy in a patient with chronic schizophrenia, resulting in significant symptomatic and functional recovery. The ethical and legal ambiguities surrounding covert medication in Japan present considerable challenges [6]. Our case exemplifies a common pattern in which family caregivers, burdened by a loved one’s non-adherence [12], resort to covert medication with the implicit involvement of prescribing physicians.

A critical aspect of this case was understanding the patient’s reasons for non-adherence. His refusal stemmed not from side effects but from poor insight [13] and the psychological burden of daily medication, which he stated “feels like admitting illness.” The LAI regimen, by removing this daily reminder, helped overcome this psychological hurdle.

Our therapeutic strategy was guided by the principles of personal recovery and shared decision-making [14,15]. By first exploring the patient’s life goals before, we framed the LAI not merely as a medical necessity but as a tool to help achieve his aspirations. This approach fostered a strong therapeutic alliance and respected his autonomy. Recent evidence suggests that LAIs improve symptom control and subjective QoL [16]. Our strategy was further supported by multidisciplinary care, including visiting nurses, as recommended in current guidelines [17].

The clinical improvements were robust, as measured by the BE-PSD [11] and GAF. The marked improvement in scores correlated with substantial functional gains. These gains align with research showing that personal and contextual factors are central to real-world functioning in schizophrenia [18].

This case offers a unique perspective within the Japanese cultural context. Family involvement in psychiatric care is deeply embedded, often leading to covert medication [19]. The LAI served as a functional “bridge,” respecting the father’s intent to ensure treatment while empowering the patient to assume ownership of his care. It allowed the father to step back from the burdensome role of medication supervisor to that of a supportive family member, reducing caregiver strain, which is known to intensify with age [12]. This outcome aligns with recent meta-analyses demonstrating the superiority of LAIs over oral antipsychotics in preventing relapse (pooled hazard ratio 0.70, 95% confidence interval 0.63-0.77) [20]. The transition to a three-monthly formulation further enhanced convenience while maintaining clinical stability, supporting the utility of ultra-long-acting formulations [16].

This single case report has certain limitations. The improvements were likely multifactorial, reflecting the combined impact of LAI initiation, hospitalization, and psychosocial support. The absence of standardized caregiver burden or QoL scales is a methodological limitation. Nonetheless, this report offers valuable insight into resolving a common ethical dilemma in schizophrenia care within the Japanese healthcare context.

## Conclusions

LAI antipsychotics, when combined with shared decision-making and a focus on personal recovery, represent an effective and ethically sound alternative to involuntary or covert medication in schizophrenia. This patient-centered approach can foster a stronger therapeutic alliance, enhance autonomy, and yield significant improvements in both clinical symptoms and real-world functioning, while reducing caregiver burden. This case suggests that clinicians facing similar ethical dilemmas should proactively explore patients’ personal recovery goals and consider LAI therapy as a key strategy to facilitate shared decision-making and resolve covert medication practices. This case presents a practical model for addressing complex treatment dilemmas in challenging sociocultural contexts and underscores the value of LAIs in modern psychiatric care.

## References

[1] (2022). Global, regional, and national burden of 12 mental disorders in 204 countries and territories, 1990-2019: a systematic analysis for the Global Burden of Disease Study 2019. Lancet Psychiatry.

[2] Charlson FJ, Ferrari AJ, Santomauro DF (2018). Global epidemiology and burden of schizophrenia: findings from the Global Burden of Disease Study 2016. Schizophr Bull.

[3] Yaegashi H, Kirino S, Remington G, Misawa F, Takeuchi H (2020). Adherence to oral antipsychotics measured by electronic adherence monitoring in schizophrenia: a systematic review and meta-analysis. CNS Drugs.

[4] Hegde PR, Gowda GS, Vajawat B, Basavaraju V, Moirangthem S, Naveen Kumar C, Bada Math S (2023). Study on covert administration of medications practices among persons with severe mental illness: a cross-sectional study. Int J Soc Psychiatry.

[5] World Health Organization (2005). WHO Resource Book on Mental Health, Human Rights and Legislation.

[6] Kimura S (2019). A literature review on the current status and ethical issues of covert medication in psychiatric care. Otemae University Journal.

[7] Saito T (2013). Hikikomori. Adolescence Without End. https://www.upress.umn.edu/book-division/books/hikikomori.

[8] (2023). Cabinet Office, Government of Japan. Survey on the awareness and daily life of children and young people [In Japanese]. Japan: Kodomo/wakamono no ishiki to seikatsu ni kansuru chōsa [Survey on the Awareness and Daily Life of Children.

[9] Correll CU, Kim E, Sliwa JK (2021). Pharmacokinetic characteristics of long-acting injectable antipsychotics for schizophrenia: an overview. CNS Drugs.

[10] Oguchi Y, Miyake N, Ando K (2024). Barriers to long-acting injectable atypical antipsychotic use in Japan: Insights from a comparative psychiatrist survey. Neuropsychopharmacol Rep.

[11] Takeuchi H, Fervaha G, Lee J, Agid O, Remington G (2016). A preliminary examination of the validity and reliability of a new brief rating scale for symptom domains of psychosis: Brief Evaluation of Psychosis Symptom Domains (BE-PSD). J Psychiatr Res.

[12] Sustrami D, Yusuf A, Fitryasari R, Suhardiningsih AV, Arifin H (2023). Determinants of burden in family caregivers of individuals with schizophrenia: a systematic review. J Psychosoc Nurs Ment Health Serv.

[13] David AS (1990). Insight and psychosis. Br J Psychiatry.

[14] Slade M, Amering M, Farkas M (2014). Uses and abuses of recovery: implementing recovery-oriented practices in mental health systems. World Psychiatry.

[15] Fujii C (2018). Policy making for recovery from schizophrenia. Journal of Mental Health.

[16] Brasso C, Bellino S, Bozzatello P, Montemagni C, Rocca P (2017). Role of 3-monthly long-acting injectable paliperidone in the maintenance of schizophrenia. Neuropsychiatr Dis Treat.

[17] Japanese Society of Neuropsychopharmacology (2025). Japanese Society of Neuropsychopharmacology: guideline for pharmacological therapy of schizophrenia. Neuropsychopharmacol Rep.

[18] Galderisi S, Rossi A, Rocca P (2014). The influence of illness-related variables, personal resources and context-related factors on real-life functioning of people with schizophrenia. World Psychiatry.

[19] Srinivasan TN, Thara R (2002). At issue: management of medication noncompliance in schizophrenia by families in India. Schizophr Bull.

[20] Kishimoto T, Hagi K, Kurokawa S, Kane JM, Correll CU (2021). Long-acting injectable versus oral antipsychotics for the maintenance treatment of schizophrenia: a systematic review and comparative meta-analysis of randomised, cohort, and pre-post studies. Lancet Psychiatry.

